# When Pain Puts Your Heart in the Fast Lane: A Case of Ventricular Tachycardia Induced by Pain From Rib Fracture

**DOI:** 10.7759/cureus.90768

**Published:** 2025-08-22

**Authors:** Mazhed Kheyrbek, Yazan Alkehef, Yusuf Alalwan, Nawar Mohamad, Nancy Mesiha

**Affiliations:** 1 Department of Cardiovascular Medicine, Henry Ford St. John Hospital, Detroit, USA

**Keywords:** arrhythmogenic cardiomyopathy, cardiac electrophysiology, idiopathic ventricular tachycardia, ventricular arrhythmia, ventricular tachycardia (vt)

## Abstract

Repetitive monomorphic ventricular tachycardia (RMVT) is the most common form of idiopathic ventricular tachycardia. It usually happens in patients with no history of cardiac disease. Many triggers have been described in the literature, specifically high catecholamine states such as surgery or acute illness. Here, we present a young patient with no past medical history who presented with acute onset right-sided upper back pain. An electrocardiogram (ECG) in the emergency room showed a wide QRS complex tachycardia with a left bundle branch morphology. The echocardiogram was significant for a reduced ejection fraction (EF) with severe global hypokinesis. She was evaluated by electrophysiology, who recommended initiating metoprolol tartrate, as the working diagnosis was thought to be RMVT. She underwent cardiac catheterization, which showed non-obstructive coronary artery disease (CAD). Upon further evaluation, she was found to have right-sided rib fractures that were not seen on the initial workup. Pain from the rib fracture was thought to be the triggering factor for her arrhythmia. Her tachycardia was resolved with beta blockers. The patient was discharged in a stable condition with electrophysiology follow-up for considering ablation.

## Introduction

Idiopathic ventricular tachycardia (VT) is a rare form of VT that happens in patients with no evidence of heart disease [1]. The most common form of this VT is repetitive monomorphic ventricular tachycardia (RMVT), comprising around 60% to 70% of all cases [1]. The origin of this VT has been located at the ventricular outflow tracts [2]. RMVT is characterized by frequent ventricular ectopy and sudden episodes of non-sustained ventricular tachycardia (NSVT). Symptoms can vary from palpitations to chest pain and syncope [1]. It usually happens at rest but can be induced by stressful events such as surgery and other illnesses [1]. Prognosis is usually favorable, but it is important to differentiate this benign VT from other causes of VT that may lead to sudden cardiac death, as management will vary greatly [3]. In this article, we present a case of RMVT induced by pain in the setting of a rib fracture, which was successfully treated with medical management.

## Case presentation

**Figure 1:**
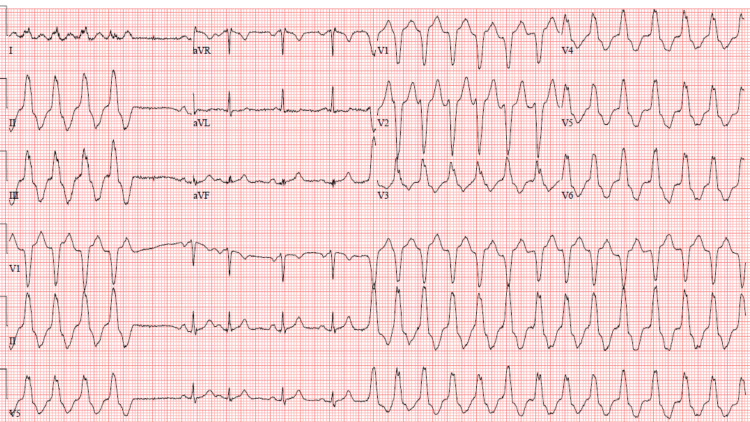
Electrocardiogram (ECG) on admission Electrocardiogram (ECG) on admission showing regular wide complex tachycardia with left bundle branch morphology and inferior axis consistent with outflow tract ventricular tachycardia.

**Figure 2:**
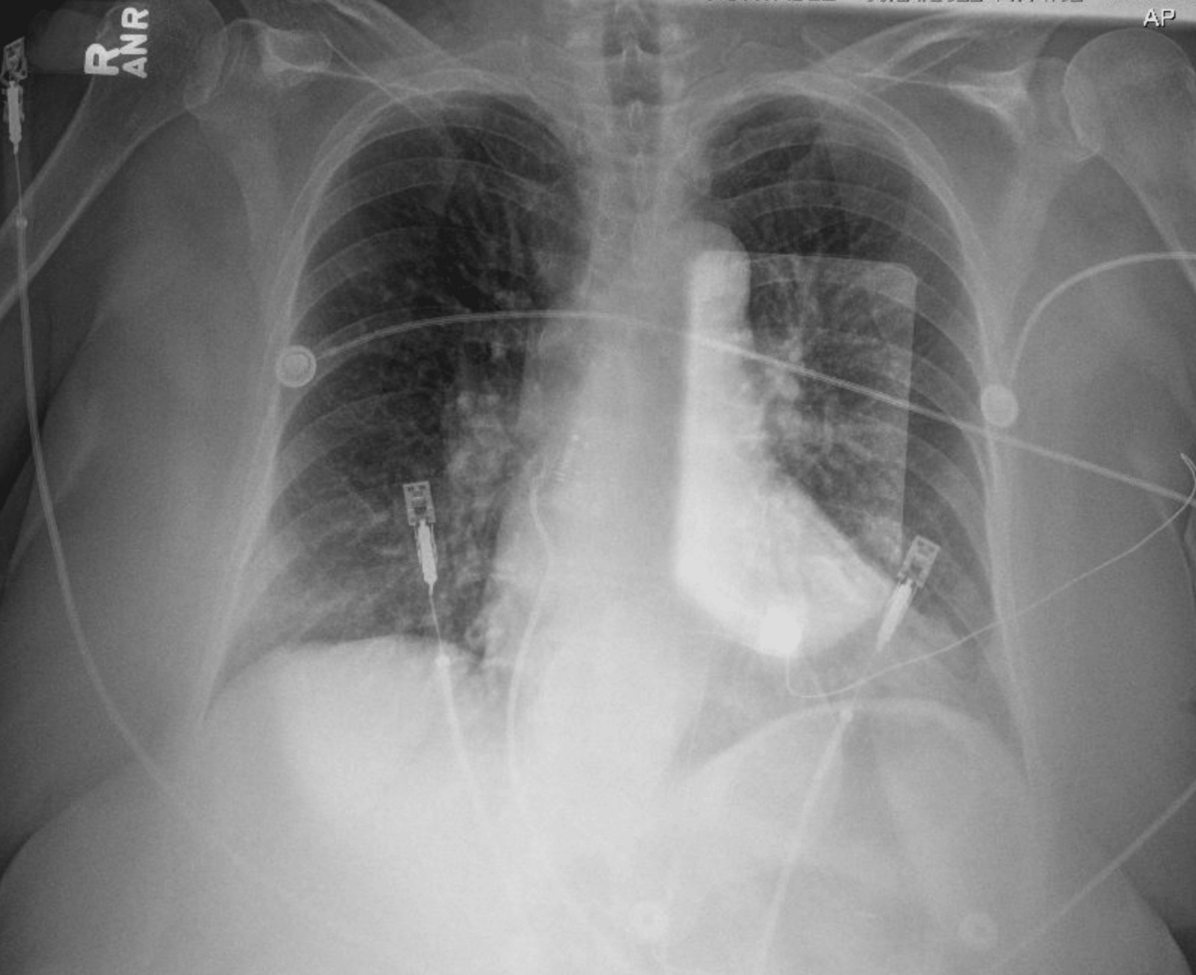
Chest X-ray (CXR) on admission CXR on admission which shows no acute cardiopulmonary findings

**Figure 3:**
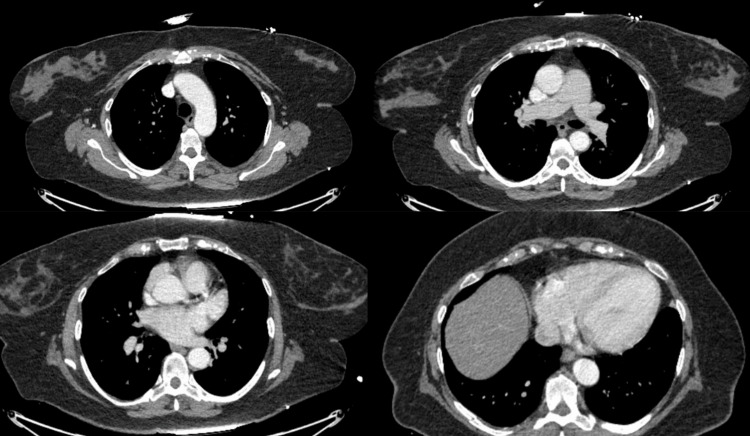
Computed tomography with angiogram (CTA) of the chest on admission CTA of the chest was negative for any acute findings like pulmonary embolism or aortic dissection.

A 62-year-old female with no past medical history presented for acute onset right-sided upper back pain that started the day prior to admission. The patient mentioned that this pain started following a fall she had after she tripped on a carpet. She described the pain as sharp in nature and worsened by movement. It was also associated with nausea and vomiting. She denied any shortness of breath, fevers, or chills. Her vitals on presentation were blood pressure: 138/101, heart rate: 157, she was afebrile, and she was saturating at 97% on room air. On physical exam, she was in acute distress with right upper quadrant (RUQ) tenderness. Her cardiac and pulmonary exams were unremarkable. Lab work showed increased white blood cell (WBC) count, high hemoglobin (Hgb) levels, normal potassium levels, borderline low magnesium, normal creatinine, and negative troponin (Table 1). Electrocardiogram (ECG) in the emergency room showed a wide QRS complex tachycardia (rate 160 bpm) (Figure 1). Chest X-ray showed no acute process (Figure 2). Computed tomography with angiography (CTA) of the chest was done and was negative for dissection or pulmonary embolism (Figure 3). The echocardiogram was significant for an ejection fraction (EF) of 20%-25% with severe global hypokinesis.

**Table 1:** Lab work on admission WBC: White blood cell, Hgb: Hemoglobin, Plts: Platelet

Test Name	Value	Reference Value
WBC	14.66 K/mcL	6 – 8 K/mcL
Hgb	17.9 gm/dL	14 – 15 gm/dL
Plts	379 K/mcL	200 – 400 K/mcL
Creatinine	0.85 mg/dL	0.70 - 1.50 mg/dL
Potassium	4.5 mmol/L	3.5 - 5.4 mmol/L
Magnesium	1.6 mEq/L	1.3 - 1.9 mEq/L
Hs-Troponin	10 ng/L	<12 ng/L

The patient received a 150 mg IV amiodarone bolus and infusion. She was evaluated by electrophysiology, who recommended discontinuing amiodarone and initiating metoprolol tartrate, as this was suspected to be outflow tract tachycardia. QRS morphology was similar to a left bundle branch (LBBB) at V1. The R/S transition point was present in leads V2-3. A big R wave was noted in the inferior leads consistent with outflow origin. Overnight, the patient was still in pain despite receiving narcotics and was having frequent episodes of wide complex tachycardia. She was then transferred to the intensive care unit, and lidocaine was started with subsequent resolution of ventricular tachycardia (VT).

**Figure 4:**
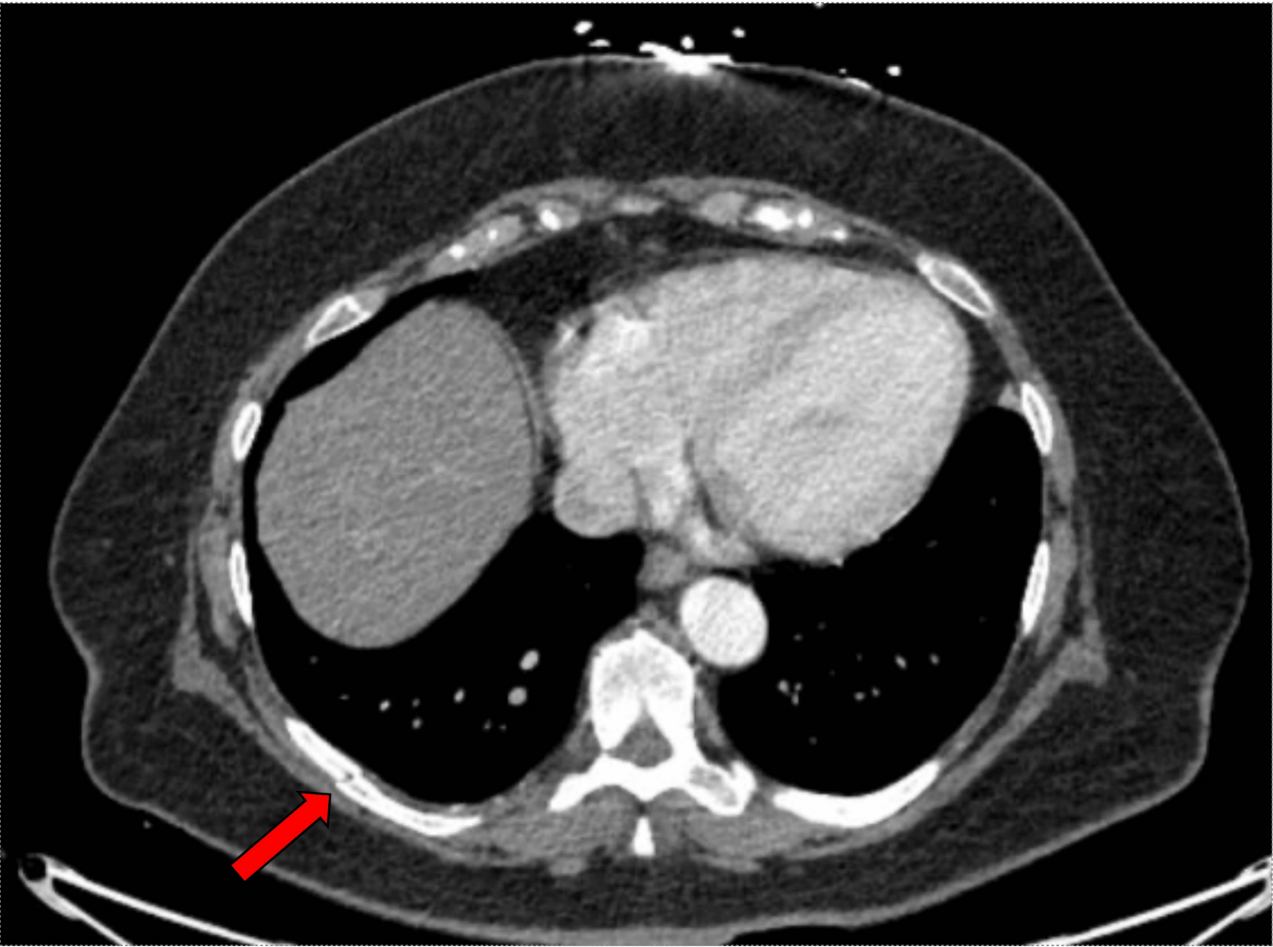
CTA of the chest on admission A CTA of the chest shows an acute fracture in the right ninth rib. CTA: Computed tomography angiography

The following day, she underwent cardiac catheterization, which showed nonobstructive coronary artery disease (CAD). Upon further evaluation of the patient’s CTA, she was found to have right-sided ninth and tenth rib fractures, which were not seen on chest X-ray (Figure 4).

A repeat echocardiogram showed improvement in EF to 60%. The patient was started on the Lopressor s50 mg twice daily, Losartan 25 mg daily, and Mexiletine 150 mg twice daily. She was stable for discharge the following day with plans for outpatient follow-up for consideration of ablation.

## Discussion

Idiopathic VT without structural heart disease is present in 10% of patients presenting with VT [4]. Outflow tract tachycardia is the most common form of idiopathic VT, comprising 70% of cases. It is characterized by a left bundle-branch block in the precordial leads. The axis is usually inferior [5,6]. Contrary to other forms of idiopathic VT, it can occur at rest. It is usually not sustained [1]. These patients also frequently have isolated premature ventricular contractions (PVCs) that match the tachycardia morphology [5]. The female population is more affected than males; the usual ages vary between 30 and 50 years [5]. There have been a few case reports of this benign VT occurring after surgery, asthma exacerbation, or, as in our case, pain [5]. The etiology of these arrhythmias is thought to be secondary to stress, which increases sympathetic and hormonal activity [6,7]. The exact mechanism of this VT is still not well understood. Lerman et al. showed that the etiology is catecholamine-mediated delayed afterdepolarizations [5]. Triggered activity has been suggested as a potential mechanism as well [5]. The activation of cyclic ampere (AMP) causes the release of calcium from the sarcoplasmic reticulum; this may explain why adenosine is successful in aborting this tachycardia [8,9]. Symptoms can vary from chest pain, palpitations, dizziness, and syncope. About 40% of the time, it may be asymptomatic, such as with our patient [5]. Most idiopathic VT syndromes have a favorable prognosis. The risk of sudden cardiac death is usually very low. However, one key point is to distinguish it from arrhythmogenic right ventricular cardiomyopathy (ARVC), as that carries much worse outcomes. ARVC usually has T wave abnormalities in right-sided precordial leads, polymorphic VT, and evidence of right ventricle structural disease [5]. In terms of treatment, the burden and acuity of symptoms usually dictate the urgency of management. It is very uncommon that this VT may lead to tachycardia-induced heart failure, which usually resolves after treatment [5]. Indeed, our patient's ejection fraction on presentation was reduced only to improve quickly after treating her VT. Management is usually comprised of medications and ablation. Acutely, VT can be terminated with adenosine, verapamil, vagal maneuvers, and lidocaine. For long-term management, beta-blockers and calcium channel blockers are considered essential [5,10]. Catheter ablation of outflow tract VT has been associated with greater than 90% success rate with very low rates of recurrence [1].

## Conclusions

In conclusion, ventricular outflow tract VT is a benign condition that happens in patients with no history of structural heart disease. It is usually induced by stressful events such as surgery or acute illness. It is known to be sensitive to adenosine, beta-blockers, and calcium channel blockers. Our patient presented with outflow tract tachycardia thought to be induced by pain from a rib fracture and was successfully treated with beta blockers. Therefore, it is essential to identify certain conditions that can precipitate this VT in otherwise healthy individuals for early diagnosis and treatment. Catheter ablation is usually curative with a low risk of recurrence. Luckily, the prognosis is excellent and rarely leads to sudden cardiac death.
